# First‐ever Marburg virus disease outbreak in Equatorial Guinea and Tanzania: An imminent crisis in West and East Africa

**DOI:** 10.1002/iid3.980

**Published:** 2023-08-28

**Authors:** Olivier Sibomana, Emmanuel Kubwimana

**Affiliations:** ^1^ Department of General Medicine and Surgery, College of Medicine and Health Sciences University of Rwanda Kigali Rwanda; ^2^ Department of Dental Surgery, College of Medicine and Health Sciences University of Rwanda Kigali Rwanda

**Keywords:** Equatorial Guinea, Marburg virus disease, outbreak, Tanzania

## Abstract

The Marburg virus, which is a member of the same virus family as the Ebola virus called Filoviridae, causes the severe infectious disease known as Marburg virus disease (MVD). Previously, different outbreaks of MVD have appeared in different African countries, including Ghana, Guinea, Uganda, Angola, the Democratic Republic of the Congo, Kenya, and South Africa. For the first time, Equatorial Guinea and Tanzania are experiencing MVD outbreaks. A total of 17 laboratory‐confirmed cases of MVD and 23 probable cases have been reported in Equatorial Guinea since the confirmation of the outbreak on February 13, 2023. The first MVD outbreak in the United Republic of Tanzania was formally confirmed by the Ministry of Health on March 21, 2023. As of 22 March, there were eight cases and five fatalities (case fatality ratio [CFR]: 62.5%). Due to the facts that Ebebiyin and Nsock Nsomo districts, the affected regions of Equatorial Guinea, borders Cameroon and Gabon, and Kagera region, the affected region of Tanzania, borders Uganda, Rwanda, and Burundi, there is fear of cross‐border spread of MVD due to cross‐border migrations, and this can be a great crisis in West and East Africa. Although there are currently outbreaks of MVD in Equatorial Guinea and Tanzania, there is currently no proof of an epidemiological connection between the two outbreaks. The aim of this article is to describe MVD, describe its first outbreak in Equatorial Guinea and Tanzania, explain the efforts being used and the challenges being faced in MVD mitigation, and recommend different measures to be taken to cope with the outbreak of MVD in Equatorial Guinea and Tanzania.

## Graphical abstract

The Marburg virus causes the severe infectious disease known as Marburg Virus Disease (MVD). Previously, different outbreaks of MVD have appeared in different African countries. For the first time, Equatorial Guinea and Tanzania are experiencing MVD outbreaks. In affected regions of Equatorial Guinea and Tanzania, surveillance and detection efforts, including contact tracing and active case finding, are recommended.

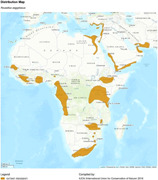

## 1 INTRODUCTION

A severe infectious disease called Marburg virus disease (MVD) is caused by the Marburg virus (MARV), a member of the Filoviridae family of viruses that also includes the Ebola virus.^1^ Hemorrhagic fever outbreaks in laboratories in Marburg and Frankfurt (in Germany), and Belgrade (in Yugoslavia [Serbia of today]), led to the discovery of the Marburg virus in 1967.^2^ Two viruses, MARV and Ravn, are members of the Marburgvirus genus, and have genetic difference of about 20%. Less genomic variation can be found in the MARV Musoke, Angola, Ci67, Ozolin, Popp, Ratayczak, and Voege variations. Both Marburg viruses are very fatal human pathogens that cause clinically similar diseases (MVDs) and have been associated with a number of hemorrhagic fever epidemics in Africa.^3^

The World Health Organization (WHO) has identified MARV as being of the utmost priority. According to WHO, The virus has an average case fatality rate that ranges from 24.0% to 88.0%,^4^ and the range can increase up to 90% depending on outbreak,^5^ showing that it is deadly and that extensive knowledge of it is required.^6^ MARV is a single‐stranded negative‐sense RNA virus that is enveloped. It is morphologically similar to silk and has a length that ranges from 800 to 14,000 nm. When it is 790 nm in length, it is most contagious. Seven structural proteins make up MARV. Although having a structure that is remarkably comparable with the Ebola virus, the MARV may cause distinct antibodies in susceptible individuals. MARV is thought to have been the first human‐discovered filovirus.^7^

**Figure 1:**
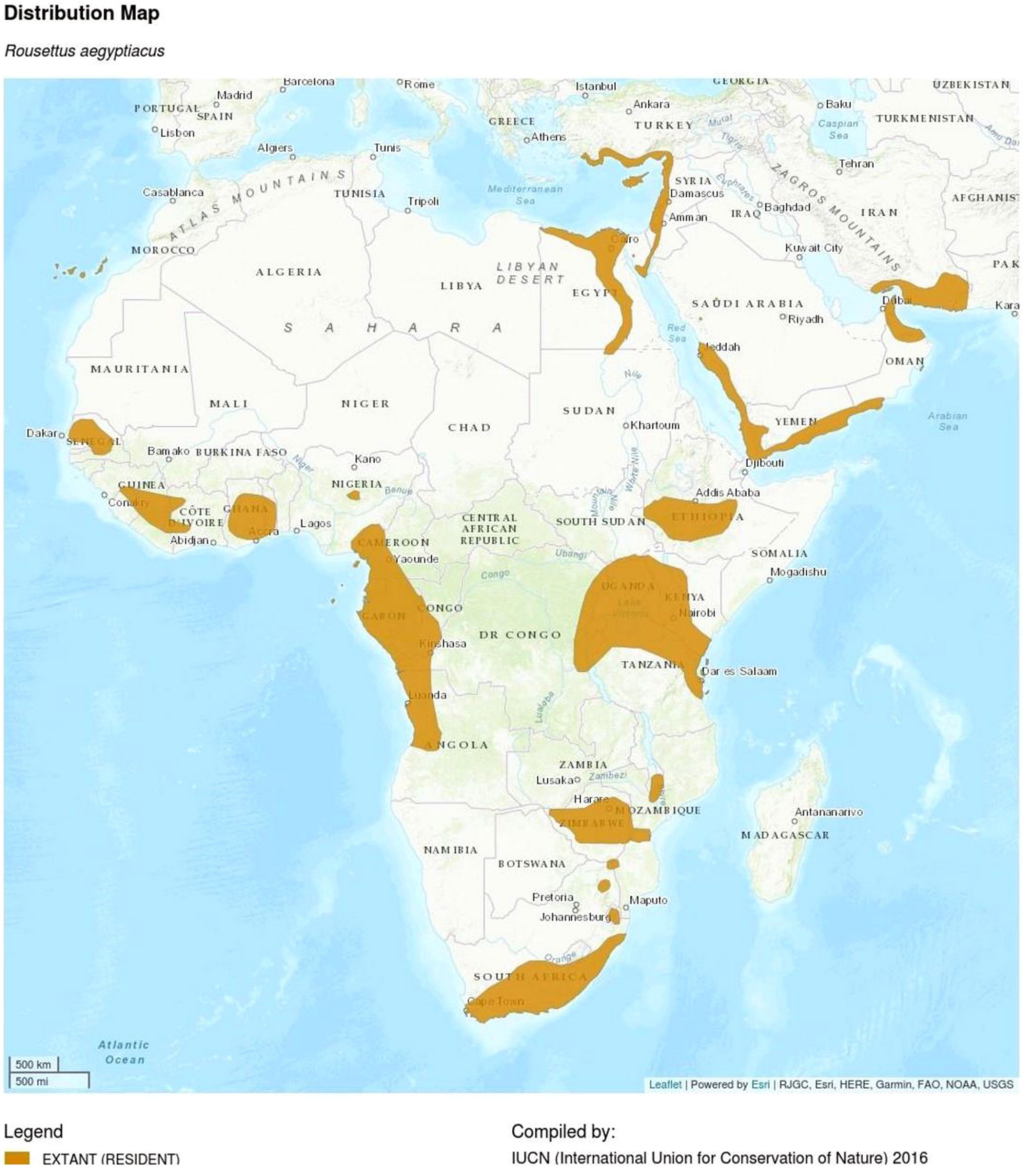
Distribution of Egyptian fruit bat (*Rousettus aegyptiacus*) in African countries. *Source*: International Union for Conservation of Nature (IUCN) 2016.

The Egyptian fruit bat (*Rousettus aegyptiacus*) serves as the reservoir of the zoonotic virus.^8^, ^9^ Previous research demonstrates that most of the primary infections associated with natural outbreaks of MARV disease to date have been related to human access to caves, for example, cave visitors and mine workers.^6^ This is because Egyptian fruit bat (*R. aegyptiacus*) are most likely to live in caves and mines. The distribution of Egyptian fruit bat (*R. aegyptiacus*) in African countries is shown in Figure 1. Following the first human‐to‐human transmission of a zoonotic disease caused by an infected animal, the disease is subsequently spread more widely via close human‐to‐human contact. This can happen either directly or by coming into contact with contaminated fomites or bodily fluids.^6^ The transmission of Marburg can also occur during burial ceremonies that involve getting into close contact with the corpse of the deceased.^10^

The incubation period of the Marburg virus lasts 3–21 days (usually between 5 and 10 days) and is probably influenced by the infectious dose and route.^11^ After the incubation period, people often have sudden illnesses with vague symptoms such as fever, chills, headache, odynophagia, myalgia, vomiting, and diarrhea. Early cases can be overlooked because they resemble more widespread infections such as malaria, typhoid, or rickettsial diseases.^12^ Early signs of MVD frequently include rash, which is characterized as nonpruritic, erythematous, and maculopapular. It starts out focally before becoming confluent and widespread. During the initial outbreak, the condition starts as a distinctly marked, pin‐sized red papule around the hair roots at the buttocks, trunk, and outside of both upper arms between the fifth and seventh day. This papule lasts up to 24 h before developing into a maculopapular rash, which later coalesce.^12^

**Figure 2:**
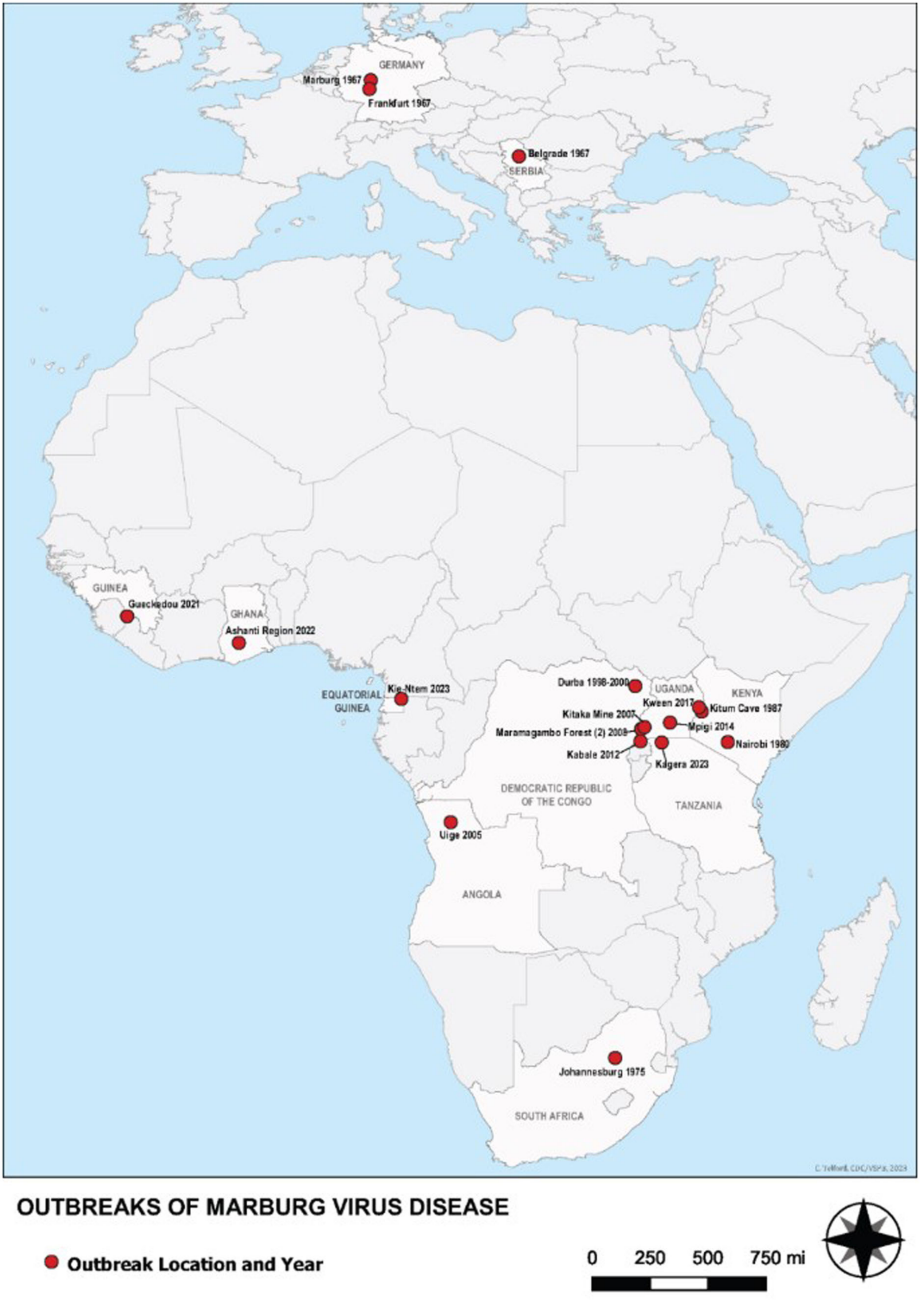
Countries reporting outbreaks of Marburg virus disease. *Source*: Centers for Disease Control and Prevention, National Center for Emerging and Zoonotic Infectious Diseases (NCEZID), Division of High‐Consequence Pathogens and Pathology (DHCPP), and Viral Special Pathogens Branch (VSPB).

Since MVD was discovered, there have been two sizable outbreaks that occurred simultaneously, one of which was linked to laboratory research involving African green monkeys that were imported from Uganda. Two unconnected sporadic occurrences of the disease occurred in 2008 in tourists from the Netherlands and the United States while they were visiting a cave in Uganda that was home to a sizable colony of Rousettus bats. Previous reports of MVD outbreaks include Ghana (2022), Guinea (2021), Uganda (2017, 2014, 2012, and 2007), Angola (2004–2005), the Democratic Republic of the Congo (1998 and 2000), Kenya (1990, 1987, and 1980), and South Africa (1975).^13^ The greatest MVD outbreak to date was in Angola in 2005, where 374 cases and 329 deaths were reported, with an 88% case fatality ratio (CFR). There have been four previous epidemics in Uganda, with case fatality rates ranging from 27% to 100% in 2007, 2012, 2014, and 2017.^14^ Table 1 shows the history of MVD outbreaks from 1967 to 2022. Countries reporting outbreaks of MVD until 2023 are shown in Figure 2.

**Table 1:** History of Marburg virus disease outbreaks from 1967 to 2022.

Year(s)	Country	Apparent or suspected origin	Reported number of human cases	Reported number (%) of deaths among cases
2022	Ghana	Ashanti Region	3	2
2021	Guinea	Guéckédou	1	1 (100%)
2017	Uganda	Kween	4	3 (75%)
2014	Uganda	Kampala	1*	1
2012	Uganda	Kabale	15	4 (27%)
2008	Netherlands ex Uganda	Cave in Maramagambo forest in Uganda, at the southern edge of Queen Elizabeth National Park	1	1 (100%)
2008	USA ex Uganda	Cave in Maramagambo forest in Uganda, at the southern edge of Queen Elizabeth National Park	1	0 (0)
2007	Uganda	Lead and gold mine in Kamwenge District, Uganda	4	1 (25%)
2004 to 2005	Angola	Uige Province, Angola	252	227 (90%)
1998 to 2000	Democratic Republic of Congo (DRC)	Durba, DRC	154	128 (83%)
1990	Russia	Russia	1	1 (100%)
1987	Kenya	Kenya	1	1 (100%)
1980	Kenya	Kenya	2	1 (50%)
1975	Johannesburg, South Africa	Zimbabwe	3	1 (33%)
1967	Germany and Yugoslavia	Uganda	31	7 (23%)

*Source*: Centers for Disease Control and Prevention, National Center for Emerging and Zoonotic Infectious Diseases (NCEZID), Division of High‐Consequence Pathogens and Pathology (DHCPP), and Viral Special Pathogens Branch (VSPB).

* Numbers reflect laboratory‐confirmed cases only.

Infectious illness epidemiology dynamics are complicated and heavily influenced by shifts in a variety of biological, demographic, environmental, and socioeconomic factors.^15^ People have been forced to invade previously uninhabited land for agricultural and mining activities due to an increasing population and the demand for resources. This could expose people to unknown pathogens, reservoir hosts and/or amplifying hosts, which greatly contribute to the transmission of viral infections, such as the Marburg virus, from animals to humans.^16^ This is corroborated by a 1997 outbreak of the Nipah virus in Indonesia. The Indonesian rainforests were burnt to make space for cultivation. Trees could not produce fruit because of the haze, which forced the local fruit bats to migrate in search of food and spread a deadly disease. Soon after the bats settled on the trees in the Malaysian orchards, the nearby pigs and pig farmers both began to get sick, perhaps from eating the fallen fruit the bats had nibbled on. 265 people experienced severe brain inflammation in 1999, and 105 died. It was the first human case of the Nipah virus, which later triggered a number of recurrent epidemics throughout Southeast Asia.^17^

As climate change is gradually occurring as a result of global warming, tropical insects can increase their habitats and spread infections to people.^18^ Although the effects of climate change on infectious diseases are poorly understood, it likely affects the habitats and densities of wildlife, which may increase the risk of human exposure to reservoir hosts or result in increased viral loads in these reservoirs, potentially increasing the frequency of disease outbreaks.^19^ The fact that infectious disease transmission patterns are being affected by climate variability and climate change is also supported by the fact that ailments commonly found in tropical and subtropical locations are spreading to new parts of the world.^20^

For the first time, Equatorial Guinea is facing MVD outbreak. The Ministry of Health and Social Welfare of Equatorial Guinea reported the first case on February 7, 2023.^10^ This is the same case in Tanzania: the country is experiencing the first outbreak of the Marburg virus disease. The MVD outbreak in the United Republic of Tanzania was declared on March 21, 2023, by the Ministry of Health (MoH) of that nation.^13^ Although there are currently outbreaks of MVD in Equatorial Guinea and Tanzania, there is currently no proof of an epidemiological connection between the two outbreaks.^13^

## 2 OUTBREAK AND EPIDEMIOLOGY OF MVD IN EQUATORIAL GUINEA

In two villages in the district of Nsock Nsomo, eastern province of Kie‐Ntem, Río Muni Region, the Ministry of Health and Social Welfare of Equatorial Guinea reported at least eight deaths that occurred between January 7 and February 7, 2023. The cases, according to the current epidemiological study, began with a fever that preceded weakness, vomiting, and blood‐stained diarrhea. In two cases, skin lesions and otorrhagia also appeared. Eight blood samples from contacts were taken on February 9, 2023, and sent to the Centre Interdisciplinaire de Recherches Médicales de Franceville (CIRMF) in Gabon, where real‐time polymerase chain reaction (RT‐PCR) testing revealed that they were free of Marburg and Ebola viruses.^10^

On February 12, 2023, eight more blood samples were obtained from different contacts and delivered to the Institute Pasteur in Dakar, Senegal. One of these samples came from a suspected case that was RT‐PCR verified to have the Marburg virus. The patient had a fever, non‐bloody vomiting, bloody diarrhea, and convulsions. On February 10, 2023, the patient passed away at the Ebebiyin District Hospital. Epidemiological evidence connected the case with four other persons of deceased cases from one of the communities in the Nsoc‐Nsomo district.^10^

**Figure 3:**
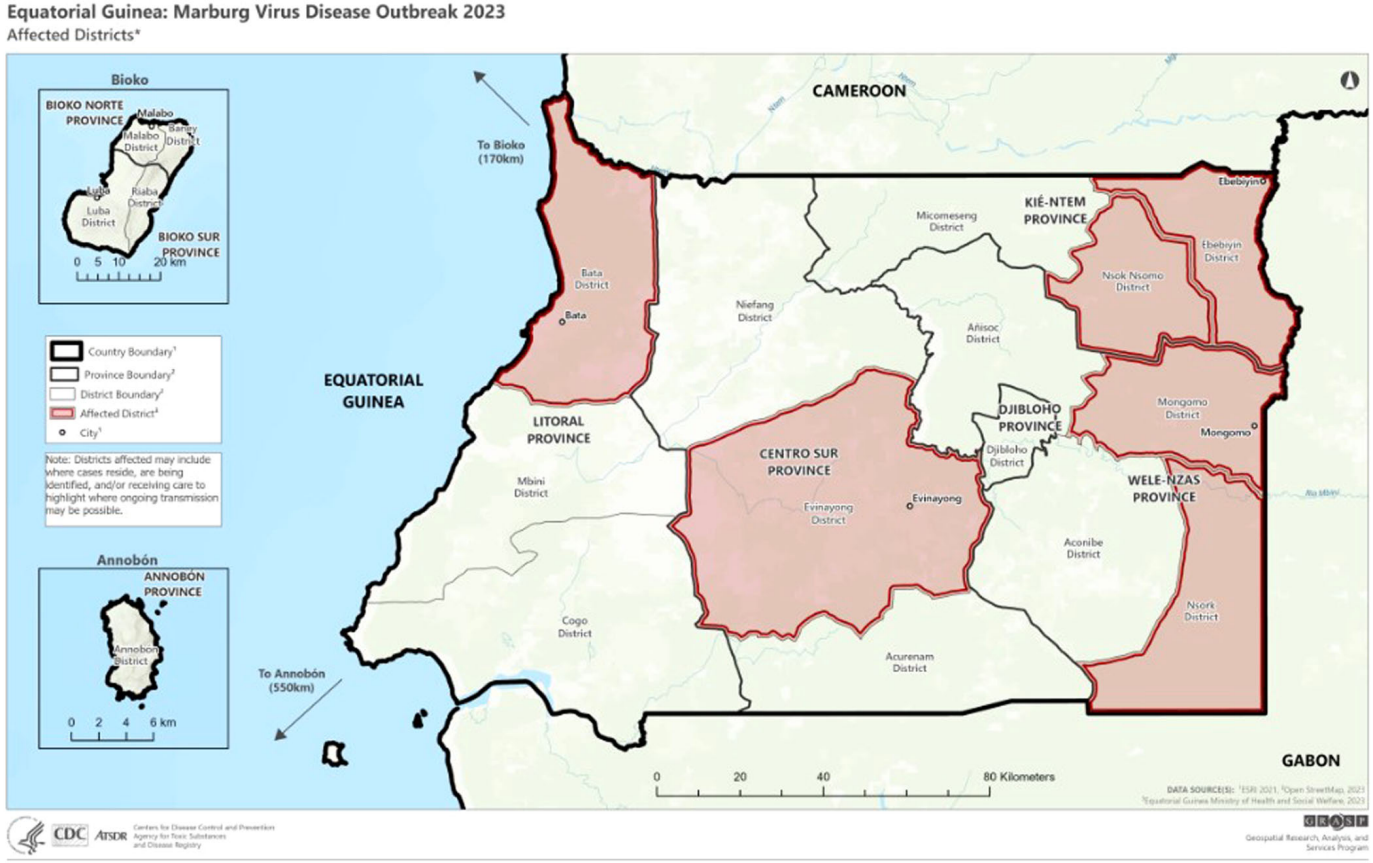
Distribution of Marburg virus disease (MVD)‐affected districts in Equatorial Guinea. *Source*: Centers for Disease Control and Prevention, National Center for Emerging and Zoonotic Infectious Diseases (NCEZID), Division of High‐Consequence Pathogens and Pathology (DHCPP), and Viral Special Pathogens Branch (VSPB).

By using RT‐PCR at a mobile lab at the Regional Hospital of Ebibeyin on March 13, 2023, samples from two more people from the Kié‐Ntem province tested positively for MVD. On March 15, 2023, a sample from a person living in Litoral province who was epidemiologically connected to a case that had been confirmed in Kié‐Ntem was positive for MVD after being subjected to RT‐PCR by the same laboratory. The two provinces (Kié‐Ntem and Litoral) are separated by around 150 km and are situated in different regions of the nation. Three other laboratory‐confirmed positive cases from the Litoral province were reported on March 18 and 20. Two further laboratory‐verified cases from the province of Centre Sur were reported on March 20. Since then, eight additional cases have been reported. The possibility of unnoticed spread of the virus in the community is suggested by the wide geographic distribution of the cases and the hazy epidemiological connections in the Centre Sur province. Since the outbreak began, a total of 17 laboratory‐confirmed cases of MVD and 23 probable cases have been reported. Out of a total of 17 confirmed cases, 12 died and all probable cases also had fatal outcomes.^21^, ^22^, ^23^ Figure 3 shows the distribution of MVD‐affected districts in Equatorial Guinea.

## 3 OUTBREAK AND EPIDEMIOLOGY OF MVD IN TANZANIA

On March 16, 2023, the Ministry of Health of the United Republic of Tanzania declared that two villages in Bukoba district, Kagera region, northern Tanzania, had seven cases and five fatalities associated with an unidentified disease. Subsequently, the Tanzanian National Public Health Laboratory used RT‐PCR to confirm the cases as Marburg virus infection. The first MVD outbreak in the nation was officially confirmed by the Ministry of Health on March 21, 2023. As of 22 March, the Kagera region had eight cases, including five fatalities (CFR: 62.5%). Treatment is still being provided to the three remaining patients. No cases have been documented outside the Bukoba district as of March 22.^13^

**Figure 4:**
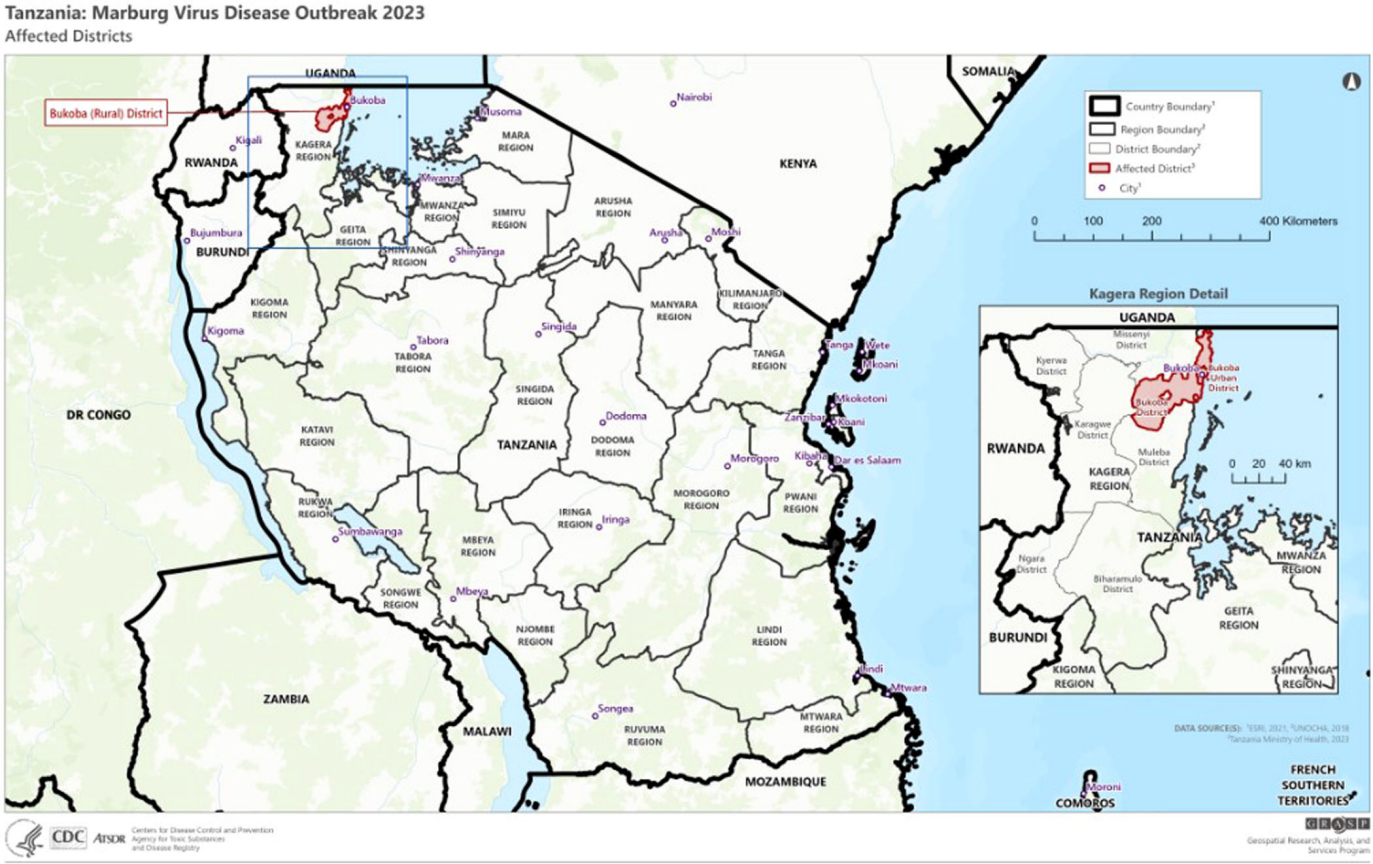
Marburg virus disease (MVD) affected district of Bukoba in Tanzania. *Source*: Centers for Disease Control and Prevention, National Center for Emerging and Zoonotic Infectious Diseases (NCEZID), Division of High‐Consequence Pathogens and Pathology (DHCPP), and Viral Special Pathogens Branch (VSPB).

The first case of MVD was identified in a person with a travel history on Goziba Island in Lake Victoria in Tanzania and symptoms were developed after the traveler returned to his village of Bukoba. The patient died in the community. Four further cases from the same family as this index case were detected. Also, among the healthcare workers who treated them, there were two cases reported, one of which resulted in death. The eighth case is still under investigation, so no information is available. Fever, diarrhea, vomiting, numerous bleeds, and kidney failure were the patients' reported symptoms. The National Public Health Laboratory tested samples from both dead and living cases and confirmed the Marburg virus.^13^ Figure 4 shows Bukoba; the MVD‐affected district in Tanzania.

## 4 EFFORTS AND CHALLENGES TO MITIGATE MVD IN EQUATORIAL GUINEA AND TANZANIA

Different public health responses are being implemented in Equatorial Guinea and Tanzania, but there are also different challenges. Within Equatorial Guinea, to identify the cause of the outbreak, comprehensive epidemiological investigations are being conducted. National teams have been sent to affected districts with the purpose of locating and isolating cases and contacts, and treating patients. The WHO has also sent out specialists in epidemiology, case management, infection control, laboratory, and risk communication to aid national response operations and guarantee community participation. Also, 500 health professionals will get a viral hemorrhagic fever kit from WHO that includes personal protective equipment in addition to tents, and tools for sample collection and analysis. As efforts are being made to establish laboratory facilities locally, WHO is providing support for the transportation of samples to labs in Senegal and Gabon.^21^

Regional and district‐level rapid response teams have been sent to Tanzania to conduct investigations and implement response plans. Contact tracking initiatives have also been implemented to keep an eye on others who exhibit comparable symptoms in the local population and medical facilities, including contacts with known patients. On March 21, health workers followed up and monitored 140 of the 161 contacts that had been discovered. In the Kagera region, risk communication initiatives have also been launched to spread messages about health awareness, education, and prevention.^13^

Although different measures have been taken against the spread of the Marburg virus in both Equatorial Guinea and Tanzania, there is still fear of the cross‐border spread of the virus. For instance, in the Equatorial Guinean districts of Ebebiyin and Nsock Nsomo, Cameroon, and Gabon, there are many cross‐border people migrations and relatively porous boundaries. This represents a risk of cross‐border spread of the virus.^21^ The affected area of Tanzania, Kagera region, borders three nations (Uganda to the north, Rwanda, and Burundi to the west), as well as Lake Victoria, and cross‐border population movements could increase the risk of disease transmission. In addition, the fruit bat species (*Roussettus aegyptiacus)* has been identified in other nearby nations that neighbor the affected Kagera region; as a result, these countries may be attached by MVD as they are home to the same bat species that carry the virus.^13^ The risk of spreading MVD in Tanzania at the national level is rated very high due to the high CFR and existing risk of the outbreak spreading to other regions of the country, the insufficient human, financial, and material resources to implement response interventions, and the likelihood that existing capacities will be overwhelmed if cases increase.^13^

For the case of Equatorial Guinea, the Equatorial Guinean government determined that its own capacity to manage epidemics and disasters was insufficient in 2022, and the government spent less than 2% of its budget on health in 2011, according to an IMF estimate, indicating underfunding of the health system in the country. Instead of prioritizing primary healthcare access and quality, health funding has a history of being concentrated on big‐scale capital projects in the field, including huge hospitals in urban areas, and according to 2017 data, there are just four doctors per 10,000 people in Equatorial Guinea. The availability of data on care seeking in Equatorial Guinea is also limited, but published studies suggest that most people tend to delay seeking treatment for febrile diseases, with rural populations and those with lower socioeconomic status being more likely to delay than others.^24^

Tanzania's MOH has highlighted various obstacles to the country's ability to respond to the MVD outbreak. These include a lack of funding, a shortage of healthcare professionals willing to treat patients, poor contact tracing tools, difficulty keeping contacts isolated, a lack of and insufficient personal protective equipment, and WASH supplies, and erroneous community perceptions. Multiple shortcomings were also found in Tanzania's MOH's 2019 assessment of Ebola readiness. The COVID‐19 pandemic, along with previous regional filovirus outbreaks and the attendant preparedness efforts, may have, however, strengthened national capacities with regard to international health regulations. For instance, the Ministry of Health in Tanzania was able to create Ebola Virus Disease contingency plan in 2019.^24^

It is difficult to diagnose Marburg virus disease. Because many other tropical febrile illnesses have some clinical symptoms of MVD in its early stages, it can be challenging to make a clinical diagnosis of the condition. Ebola virus disease, malaria, typhoid fever, leptospirosis, rickettsial diseases, and plague are a few diseases that must be ruled out.^21^ This difficulty in the diagnosis of MVD can delay the identification and treatment of a disease, the factor that can increase its mortality rate and the transmission rate.

Except rehydration with oral or intravenous fluids and treatment of specific symptoms that improve survival, no current vaccines or antiviral medications are approved to cure MVD, despite the fact that a variety of potential treatments, including blood products, immune, and drug therapies, are being evaluated.^21^ The Marburg virus (MARV) outbreak in Guinea and Ghana led to the formation of the “MARVAC” consortium, which is made up of experts in the field of vaccine research and development. The consortium's goal is to assist in a quick response to the threat posed by this infectious disease. Soon after the virus was identified, work on a MARV vaccine began with only a little progress. Several different vaccine platforms for MARV have been tested in rodent models, but only some of these candidate vaccines showed protective efficacy in nonhuman primates (NHPs). However, Currently, there are no MARV vaccines or treatments that have received regulatory agency approval.^25^

## 5 RECOMMENDATIONS

Controlling the Marburg virus disease outbreak requires a variety of interventions, including social mobilization, case management, surveillance, including contact tracking, good laboratory service, and infection prevention and control, including safe and dignified burials.^21^ In all impacted health zones, surveillance and detection efforts—including contact tracing and active case finding—should be stepped up. Identifying those who may have come into contact with someone who has the Marburg virus and tracking their health for 21 days are among the measures to be taken to control MVD outbreaks. Other measures include separating healthy and ill people to stop further transmission, caring for confirmed patients, maintaining good hygiene, and keeping the environment clean.^13^

To prevent contact with patients' blood and other bodily fluids as well as contaminated surfaces and objects, healthcare workers caring for patients with confirmed or suspected MVD should take additional infection prevention and control procedures in addition to conventional safety measures. Furthermore, educating people about the risk factors for Marburg infection and the preventive measures they can take to reduce human exposure to the virus are important steps in reducing human infections and fatalities.^13^

Furthermore, WHO advises male MVD survivors to engage in safer sexual activities and maintain good personal cleanliness for 12 months from the onset of symptoms until their semen has tested Marburg virus‐free twice. Body fluid contact should be avoided, and cleaning with soap and water is advised. WHO advises against segregating male or female convalescent patients whose blood has been tested negative for the Marburg virus.^21^

Finally, profound research studies to investigate the exact source of MARV in both Equatorial Guinea and Tanzania are needed. Although there are currently outbreaks of MVD in Equatorial Guinea and Tanzania, there is currently no proof of an epidemiological connection between the two outbreaks. Deep molecular and genetic epidemiological studies to compare MARV variant isolates from Equatorial Guinea with isolates from Tanzania are needed to explore the connection between the two outbreaks. The findings from such studies can also be important in comparing the two outbreaks with previous MVD outbreaks in different countries. This can be useful in understanding the nature of the virus and drafting evidence‐based measures to control and prevent MVD outbreaks in Africa and worldwide.

## 6 CONCLUSIONS

Equatorial Guinea and the United Republic of Tanzania are experiencing the first‐ever outbreaks of the Marburg virus. Different public health responses are being implemented in Equatorial Guinea and Tanzania, including treating patients, locating and isolating cases and contacts, case management, infection control, risk communication, and the provision of hemorrhagic fever kits to health professionals who involve in outbreak responses. The unavailability of vaccines and drugs against MVD and the fear of its cross‐border spread are major challenges. In affected regions of Equatorial Guinea and Tanzania, surveillance and detection efforts, including contact tracing and active case finding, are recommended. Furthermore, healthcare professionals dealing with suspected cases of MVD are advised to take additional infection prevention and control procedures in addition to conventional safety measures.

## AUTHOR CONTRIBUTIONS

**Olivier Sibomana**: Conceptualization; methodology; project administration; supervision; validation; writing—original draft. **Emmanuel Kubwimana**: Writing—review & editing.

## CONFLICT OF INTEREST STATEMENT

The authors declare no conflict of interest.
